# P68. A new EGFR - EpCAM bispecific antibody enhances the efficacy of adoptive T-cell therapy in a murine gastric tumour model

**DOI:** 10.1186/2051-1426-2-S2-P42

**Published:** 2014-03-12

**Authors:** S Kobold, J Steffen, S Grassmann, R Castoldi, J Schmollinger, C Sustmann, G Nierderfellner, C Klein, C Bourquin, S Endres

**Affiliations:** 1 Division of Clinical Pharmacology, Munich, Germany; 2 Roche Diagnostics GmBH, Discovery Oncology, Penzberg, Germany

## Background

A limiting step for adoptively transferred tumour-specific T cells is their recruitment from the blood circulation to the proximity of tumour cells and subsequent engagement in direct tumour cell contact. We hypothesised that a bispecific antibody recruiting T cells to a target antigen on tumour cells could enhance T-cell-tumour interaction and thus increase the efficacy of adoptively transferred T cells.

## Material and methods

A new bispecific murine IgG2a antibody (BsAb) was generated that recognises EpCAM as a tumour antigen and truncated EGFR (D-EGFR) as an inert surface marker protein on transduced T cells. T cells from transgenic mice for TCR specific for the SV40 large T antigen (TCR-1) were retrovirally transduced with D-EGFR. S.c. tumors were induced in C57Bl/6 mice by injecting mGC8 cells derived from a syngeneic large T antigen expressing EpCAM-positive gastric tumor.

## Results

*In vitro*, the BsAb increased (4-fold) binding of transduced T cells to EpCAM positive tumour cells. In the presence of the BsAb, tumour-directed T cells efficiently lysed EpCAM-positive cells (83 % at a 10:1 effector to target ratio). *In vivo*, the antibody reached EpCAM+ tumour cells as evidenced by immunofluorescence. mGC8 tumour-bearing mice were treated twice with a combination of the BsAb and transduced TCR-I T cells. Tumour growth was significantly reduced for over 30 days (n=12) compared with control groups (transduced T-cells or BsAb alone) and survival was prolonged by > 30 days (p<0.001).

## Conclusions

Co-administration of a BsAb bridging adoptively transferred tumour-specific T cells via an inert surface molecule to a tumour-associated surface antigen enhances the efficacy of therapeutic T cell transfer.

